# Neutralization of Botulinum Neurotoxin Type E by a Humanized Antibody

**DOI:** 10.3390/toxins8090257

**Published:** 2016-09-12

**Authors:** Yağmur Derman, Katja Selby, Sebastian Miethe, André Frenzel, Yvonne Liu, Christine Rasetti-Escargueil, Arnaud Avril, Thibaut Pelat, Remi Urbain, Alexandre Fontayne, Philippe Thullier, Dorothea Sesardic, Miia Lindström, Michael Hust, Hannu Korkeala

**Affiliations:** 1 Department of Food Hygiene and Environmental Health, Faculty of Veterinary Medicine, University of Helsinki, Helsinki 00014, Finland; katja.selby@helsinki.fi (K.S.); miia.lindstrom@helsinki.fi (M.L.); hannu.korkeala@helsinki.fi (H.K.); 2 Institut für Biochemie, Biotechnologie, und Bioinformatik, Technische Universität Braunschweig, Abteilung Biotechnologie, Braunschweig 38106, Germany; s.miethe@tu-braunschweig.de (S.M.); andre.frenzel@tu-bs.de (A.F.); 3 YUMAB GmbH, Rebenring 33, Braunschweig 38106, Germany; 4 Division of Bacteriology, National Institute for Biological Standards and Control (NIBSC), A centre of Medicine and Healthcare products Regulatory Agency, Blanche Lane, South Mimms, Potters Bar, Hertfordshire EN6 3QG, UK; yvonne.liu@nibsc.org (Y.L.); christine.escargueil@laposte.net (C.R.-E.); Thea.Sesardic@nibsc.org (D.S.); 5 Institut de Recherche Biomédicale des Armées (IRBA), Département des Maladies Infectieuses, Unité biothérapies anti-infectieuses et immunité, Brétigny-sur-Orge, 1 place du Général Valérie André 91220, France; arnaud.avril@irba.fr (A.A.); thibaut.pelat@biotem.fr (T.P.); pthullier@yahoo.com (P.T.); 6 LFB Biotechnologies, Therapeutic Innovation Department, 59 Rue de Trévise, Lille Cedex BP 62006-59011, France; remi.urbain@ecdysispharma.com (R.U.); fontaynea@lfb.fr (A.F.); 7 Ecdysis Pharma, Bioincubateur Eurasanté, 70 rue du Dr Yersin, Loos 59120, France

**Keywords:** botulinum neurotoxin type E, botulism, antibody

## Abstract

Botulinum neurotoxins (BoNTs) cause botulism and are the deadliest naturally-occurring substances known to humans. BoNTs have been classified as one of the category A agents by the Centers for Disease Control and Prevention, indicating their potential use as bioweapons. To counter bio-threat and naturally-occurring botulism cases, well-tolerated antibodies by humans that neutralize BoNTs are relevant. In our previous work, we showed the neutralizing potential of macaque (*Macaca fascicularis*)-derived scFv-Fc (scFv-Fc ELC18) by in vitro endopeptidase immunoassay and ex vivo mouse phrenic nerve-hemidiaphragm assay by targeting the light chain of the botulinum neurotoxin type E (BoNT/E). In the present study, we germline-humanized scFv-Fc ELC18 into a full IgG hu8ELC18 to increase its immunotolerance by humans. We demonstrated the protection and prophylaxis capacity of hu8ELC18 against BoNT/E in a mouse model. A concentration of 2.5 ng/mouse of hu8ELC18 protected against 5 mouse lethal dose (MLD) in a mouse protection assay and complete neutralization of 1 LD50 of pure BoNT/E toxin was achieved with 8 ng of hu8ELC18 in mouse paralysis assay. Furthermore, hu8ELC18 protected mice from 5 MLD if injected up to 14 days prior to intraperitoneal BoNT/E administration. This newly-developed humanized IgG is expected to have high tolerance in humans.

## 1. Introduction

The botulinum neurotoxin (BoNT) is the most potent natural toxin known, with an estimated lethal dose of 1 ng/kg to an average human [1,2]. Botulism is a neuroparalytic disease caused by BoNT, secreted by the strictly anaerobic, Gram-positive, spore-forming bacterium, *Clostridium botulinum* [3]. Strains of *C. botulinum* represent diverse genetic, metabolic, and physiologic features; hence, they are categorized into four groups (I-IV) [4,5]. To date, seven serologically different BoNT types have been identified and designated as A through G [6]. An additional BoNT serotype H was proposed [7,8] but a recent report indicates that it is rather an F/A mosaic and can be fully neutralized by anti-A antisera [9]. BoNT types A (BoNT/A), B, E, and, rarely, F, are associated with human botulism, while types C and D cause botulism in animals, whereas BoNT/G has not been associated with illness [4]. *C. botulinum* secretes BoNT during its vegetative growth with peak production occurring at the late logarithmic growth phase [10,11]. Due to extreme toxicity, Centers for Disease Control and Prevention (CDC) classified BoNTs as a Tier 1, Category A agent, emphasizing its potential for use as a bioweapon. In fact, reports suggest that several governments have stockpiled BoNT, and the Japanese cult Aum Shinrikyo have attempted to use BoNT for bioterrorism [1,12,13].

BoNTs are produced as a complex progenitor toxin [14,15]. Within the toxin complex, a single-chain BoNT molecule undergoes proteolytic activation. This activation generates a heterodimeric molecule consisting of a 100-kDa heavy chain (HC) and a 50-kDa light chain (LC) that are connected by a disulfide bond [16,17]. The HC facilitates binding of the toxin molecule with high affinity to the target receptors located on the neuronal cell surface [18]. Binding is followed by irreversible up-take of the BoNT into the neuron cytoplasm via endocytosis [19,20]. At this stage BoNT is trapped in an endosome. Acidification of endosome lumen allows translocation of the LC into the cytoplasm [21,22]. This is followed by the reduction of the disulfide bond between the HC and LC [23]. The LC of BoNT establishes its zinc-dependent protease activity by cleaving specific proteins within the SNARE complex (*N*-ethylmaleimide-sensitive fusion attachment protein receptor) and blocks their essential role in the release of the neurotransmitter, acetylcholine, into the neuromuscular junction [21,24]. This blockage results in disruption of the signal transduction and leads to flaccid paralysis of the affected muscles. Botulism is caused by BoNT reaching the peripheral nerve endings via the bloodstream.

*C. botulinum* is ubiquitous in nature, and the northern hemisphere is heavily contaminated by spores of BoNT/E producing group II strains [25,26,27,28] which has been highlighted in several outbreaks [29,30,31]. BoNT/E is produced by non-proteolytic strains that require host-provided proteolytic activation resulting in an increase in its potency [32]. BoNT/E cleaves synaptosomal-associated protein of molecular mass 25-kDa (SNAP25) at residues arginine^180^°–isoleucine^181^.

Depending on the amount of BoNT consumed, the time of botulism symptom onset may vary (12–72 h) [4]. The clinical manifestation of botulism can initially be seen at the cranial muscles as the relative blood flow is high and the innervation of the muscles in this body part is dense [33]. Double or blurred vision, difficulty in speaking and swallowing, dry mouth, and facial paralysis are characteristic symptoms of botulism. If the disease progresses, symmetrical cranial flaccid paralysis descends through the limbs. Without treatment, paralysis of the respiratory muscles may lead to death [34,35].

The treatment of botulism consists of immediate administration of antitoxin and intensive palliative care of the patient. The only specific strategy to treat botulism is to neutralize the circulating toxin with an antitoxin, thus preventing the irreversible internalization of BoNT into the neurons. The antitoxin product available for treatment of botulism in infants is a human-derived immune globulin, named BabyBIG [36]. To treat botulism in non-infant patients, an equine-derived heptavalent botulinum antitoxin (HBAT) is available through the CDC [37]. However, animal-derived antitoxin treatment may cause side effects ranging from local skin reactions to serum sickness, [38,39]. Therefore, for human application, optimal tolerance of antibodies is of major therapeutic relevance. One method to increase the immune tolerance of antibodies derived from non-human primates (NHP) is called germline-humanization. Here, the NHP antibody framework regions (FRs) were modified by a series of mutations to increase the level of identity with the human FRs encoded by the closest human germline genes [40]. It has been shown that human germline FRs of IgM antibodies are better tolerated by the immune system than FR sequences derived from IgG antibodies, which carry somatic hypermutations resulting from affinity maturation that probably form immunogenic sequences [41,42].

Due to the high similarity of NHP and human antibodies, online tools such as IMGT/V-QUEST (the International ImmunoGeneTics information system) can be used for identification of the human germline V(D)J gene segments which are most similar to the given sequence encoded by the NHP variable regions. Differences in the amino acid (AA) sequences are indicated by the Germinality Index (GI). The GI can be used as a predictor of tolerance when modifying the NHP FRs by AA exchange to increase the level of identity with FRs encoded by human germline gene segments. This method has been successfully applied in previous antibody development studies. The humanization of 35PA83, a Fab antibody of macaque origin neutralizing anthrax toxin, which was used as a benchmark for this study, led to an increase of the GI from 87.6% up to 97.7%, a value higher than that of the fully-human Fab 83K7C (GI 91.9%) [43]. Furthermore, the antibody WO-2 against Aβ peptide, associated with Alzheimer's disease, was successfully humanized by germline humanization, retaining its affinity and ability to inhibit aggregation and oligomer-mediated toxicity [44].

In our previous study, a macaque (*Macaca fascicularis*) was immunized by injection of recombinant LC of BoNT/E to construct an immune phage display library [45]. Subsequently, a panel of single-chain Fragment variables (scFv) were isolated and used to inhibit the endopeptidase activity of BoNT/E and to neutralize BoNT/E-induced paralysis in ex vivo mouse phrenic nerve-hemidiaphragm assays [45,46]. The scFv-Fc that showed the most effective neutralization activity (ELC18) was selected and shown to protect mice from 1LD50 BoNT in the non-lethal mouse flaccid paralysis assay at 1.6 ng/dose [45]. In this study, the level of identity of ELC18 FRs with FRs encoded by the human germline gene sequences were increased, described as germline-humanization, in order to develop potent IgGs against BoNT/E with putative high tolerance by the human immune system [40]. We showed high in vivo neutralization activity and protection capacity of the newly-developed humanized IgG derived from ELC18 against BoNT/E LC in a mouse model. This antibody, when combined with previously reported four antibodies targeting HC and LC of BoNT/A and BoNT/B [47] will provide promising candidate for further development of an oligococktail of protective recombinant super-humanized IgGs against all three major botulinum toxins.

## 2. Results

### 2.1. Comparison between scFv ELC18 and Human Germline Genes

**Figure 1:**
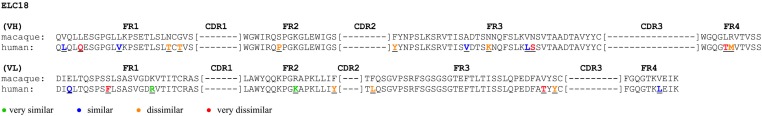
Sequence of the macaque framework regions and those coded by the most similar human germline genes. Based on the physiochemical classes of the amino acids (AA), differences in the framework regions are classified into very similar AA (green), similar AA (blue), dissimilar AA (orange), and very dissimilar AA (red).

To compare ELC18 to human germline genes, we performed analysis using the IMGT/V-QUEST tool. For VH, the identified V, D, and J gene segments were IGHV4-28*01, IGHD2-21*01, and IGHJ3*01, respectively. For VL, the V and J gene segments were IGKV1-9*01 and IGKJ2*03, respectively. The GI for VH and VL were calculated using IMGT/DomainGapAlign and provided an indication of the identity between framework regions of ELC18 and those encoded by the most similar human germline genes, as a percentage. The GI-value for VH was 85.7% and for VL 89.9%. Furthermore, we located and evaluated the differences in sequence between ELC18 framework regions and those coded by the ELC18 most similar human germline genes. In total, 22 of the 180 residues of the eight framework regions differed between ELC18 and those of the selected human germline gene segments (Figure 1 and Table 1).

The GIs of the framework regions are shown in Figure 1. Based on the physiochemical characteristics of AAs, differences in the FRs were categorized (Figure 1). For VH, we identified four residues classified as similar AA, six residues as dissimilar AA, and three residues as very dissimilar AA (Figure 1). For VL, two residues were classified as very similar AA, two as similar AA, three as dissimilar AA, and two as very dissimilar AA, respectively (Figure 1).

### 2.2. Humanization of the Macaque scFv ELC18 by Germline-Humanization

**Figure 2:**
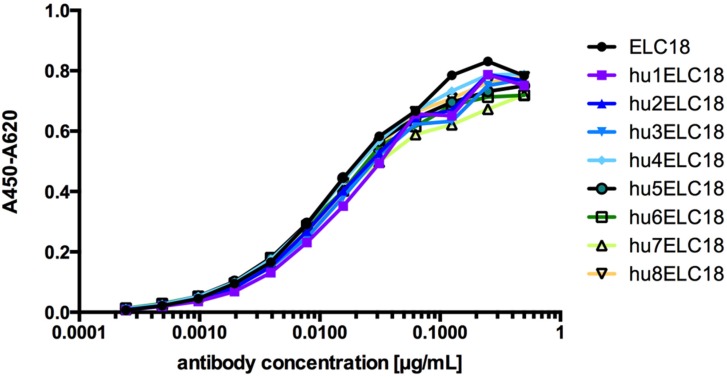
ELISA assay of all humanized ELC18 variants (from hu1ELC18 to hu8ELC18) and non-humanized ELC18 (No reactivity against BSA, not shown).

The alignment of ELC18 and the human germline genes was done by using the IMGT/V-QUEST online tool to identify the human genes that are most similar to the variable regions of ELC18 to calculate the GI. For the humanization process of ELC18 we used a multistep approach. In the first step, we designed humanized variants of the variable domains of ELC18 by exchanging AAs in the FRs that differ from the human germline sequence with their human counterpart classified as very similar and similar AA. The resulting variable domains were called hu_1_VH and hu_1_VL. In the next step, we replaced the AA classified as dissimilar AA, resulting to the humanized variants hu_2_VH and hu_2_VL and combined each variable domain with each other including the parental VH and VL. By exchanging this AA, we were able to increase the GI value of the humanized antibodies up to 97.3% (hu8ELC18) (Table 2). To validate the quality of the humanization process, we produced eight distinct variants as scFv-Fc antibodies and the antigen binding of the eight humanized variants, and the parental ELC18 was compared and validated by ELISA using an immobilized BoNT/E light chain (Figure 2). No significant difference in the antigen binding was observed between the humanized variants and the parental ELC18 (no reactivity against BSA, data not shown). For hu8ELC18, only five of the very dissimilar AAs were retained and not replaced by the human counterparts (Figure 1). For comparison, the average GI of 500 scFvs isolated from the naive human antibody gene library, HAL7/8 was 96.8% (VH), 95.4% (VL lambda) and 94.8% (VL kappa). With the highest GI value of 97.3% of all ELC18 variants, hu8ELC18 was selected for further in vivo studies. Therefore, scFv-Fc hu8ELC18 was re-cloned and produced as germline-humanized IgG.

### 2.3. Mouse Protection Assay Performed with IgG hu8ELC18

The in vivo protective capacity of the humanized IgG hu8ELC18 was determined by mouse bioassay against BoNT/ E toxin complex. At concentration of 2.5 ng/mouse hu8ELC18 antibody fully protected against 5 MLD of the activated-BoNT/E complexes. The results of the in vivo protection assays of the different hu8ELC18 concentrations against a test dose of 5 MLD of BoNT/E complexes are summarized in Table 3. None of the mice showed botulism-specific symptoms during the entire four days of the assays. Mice given toxin alone in the absence of antibody were all showing typical systemic symptoms of botulinum intoxication after 3–5 h post injection.

### 2.4. Mouse Paralysis Assay Performed with IgG hu8ELC18

**Figure 3:**
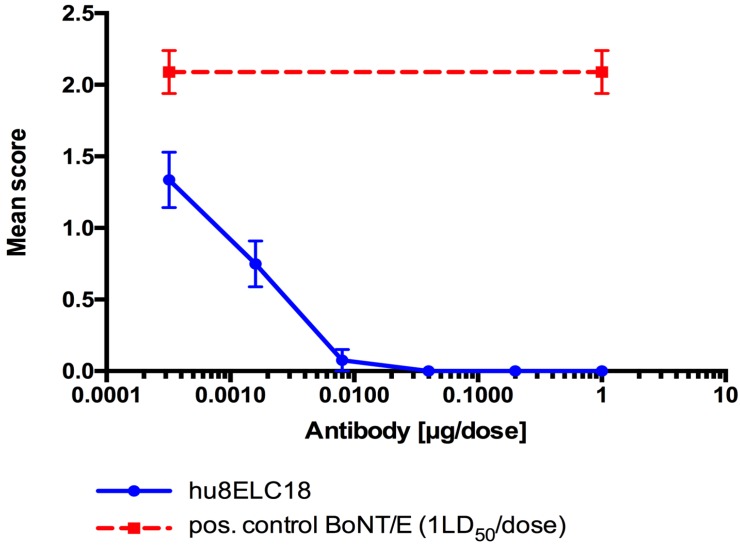
Neutralization activity of hu8ELC18 in mouse flaccid paralysis. Pure BoNT/E3 toxin (1.0 LD50 per dose) was pre-mixed with a range of antibody concentration (from 1.0 µg per dose). Antibody-toxin mixtures were left for 30 min at room temperature before injecting subcutaneously 0.1 mL (*n* = 4) into the left inguinocrural region of female, MF1 strain of mice. Animals were scored at 24 h post injection. Results are expressed as mean score of 4 mice ± SEM. The positive control group of mice (*n* = 4) was injected with 1 LD50 of BoNT/E3 toxin alone, and the negative control group (*n* = 2) were given 1.0 µg of antibody in the absence of toxin (data not shown).

Neutralization activity of IgG hu8ELC18 was also assessed in vivo paralysis model with a range of concentrations of antibody (from 1.0 µg to 0.32 ng per dose) and pure BoNT/E3 toxin. In this assay, a sub-lethal dose of toxin was subcutaneously injected into the left inguinocrural region of mice to induce local flaccid paralysis. The results from in vivo neutralization in the mouse flaccid paralysis model against pure BoNT/E3 (1.0 LD50 per dose) recorded at 24 h post injection are represented in Figure 3. Complete neutralization of 1 LD50 of pure BoNT/E toxin was achieved with 8 ng of hu8ELC18, and partial neutralization with two further five-fold dilutions (1.6 and 0.32 ng). Animals injected with 1.0 µg of antibody in the absence of toxin did not show any sign of abnormality (data not shown). Mice injected with toxin alone, in the absence of antibody, developed visible abdominal ptosis at the site of injection, as a result of flaccid paralysis.

### 2.5. Prophylaxis Assays Performed with IgG hu8ELC18

We tested the prophylactic capacity of IgG hu8HLC18 in a mouse model. Each mouse received 25 µg of hu8HLC18 by i.p. route on day 1, 3, 7, and 14 before administration of BoNT/E complex. The hu8ELC18 at a concentration of 25 µg/mouse protected mice challenged with a test dose of 5 MLD/mouse BoNT/E complex if administered (i.p.) up to 14 d in advance. All mice survived during the four days post challenge (Table 4). None of the mice showed botulism specific symptoms during the assays. Mice given toxin alone in the absence of antibody were all showing typical systemic symptoms of botulinum intoxication after 3–5 h post injection.

## 3. Discussion

The anti-BoNT/E IgG hu8ELC18 developed in this study showed high neutralization capacity. At a concentration of only 2.5 ng IgG/mouse, IgG hu8ELC18 protected mice fully against 5 MLD/mouse BoNT/E. Furthermore, studies in paralysis model support this finding as 8 ng of hu8ELC18 completely protected mice against paralysis induced by 1 LD50 of pure BoNT/E3 and some protection was also observed at concentrations as low as 0.32 ng of hu8ELC18. In addition, hu8ELC18 showed protection in the mouse model when administered up to 14 days prior to BoNT/E challenge. The three-fold difference in potency between the paralysis and protection assays could be explained by use of toxins obtained from different *C. botulinum* strains in the two assay models. Whereas crude BoNT/E obtained directly from the culture supernatant was used in the protection test, pure toxin was used in the paralysis model. Furthermore, the two toxin preparations were calibrated in different laboratories.

In our previous studies, within the AntiBotABE project, the macaque scFv-Fc ELC18 was isolated from an immune library neutralizing BoNT/E toxicity in the ex vivo mouse phrenic nerve-hemidiaphragm assay that mimics the in vivo respiratory paralysis caused by BoNT/E [45]. Furthermore, ELC18 was highly effective in the in vivo mouse flaccid paralysis assay. Full protection against pure BoNT/E (1.0 LD_50_ per mouse) was achieved with 1.6 ng scFv-Fc antibody [45]. Previous related studies with the chimeric antibody lumiliximab, consisting of the variable regions of a macaque in combination with the human constant regions, showed good immune tolerance in humans; thus, the hu8ELC18 is predicted to have also high tolerance in humans because of its high GI [46,47,48].

The humanization of hu8ELC18 provides a potentially promising method to develop antibodies against BoNT for human therapy against botulism. Furthermore, germline-humanization may increase immune tolerance of these newly developed antibodies [40]. This method was successfully used for the humanization of 35PA838, an antibody neutralizing the anthrax toxin, currently in pre-clinical development which was isolated from a macaque immune library and for which we increased its GI value to 97.8% [43]. For comparison, the average GI value calculated out of 500 scFvs isolated from the human naive antibody gene library HAL7/8 [49] was 95.7%. The number of differences in AAs between the FR regions of the macaque ELC18 and those encoded by the closest human germline genes was 22, resulting in a GI value of 87.8%. These differences could either correspond to somatic hypermutations or to differences between the macaque and human germline genes, and both could be immunogenic. Therefore, we intended to increase the GI value using a systematic approach. We adapted the FRs of ELC18 to their human IgM counterparts. The human germline FRs as part of IgM antibodies are expected to be as well tolerated as any other human protein, in contrast to the FR sequences derived from IgG antibodies, which carry somatic hypermutations resulting from affinity maturation and potentially form immunogenic sequences [40,41,42].

The medical countermeasures against human botulism primarily consist of a prophylactic vaccination of people with a high risk of exposure (e.g., first responders, scientists, troops) and specific therapy with neutralizing antibodies. The toxoid vaccines require administration well in advance to exposure to BoNT because they produce slow immunity; hence, the efficacy of such vaccines is limited [50]. In addition, vaccines have the disadvantage of limiting the future therapeutic use of BoNTs for the treatment of several diseases such as some neuromuscular disorders [51]. Although development of vaccines against BoNTs started more than 60 years ago, no licensed vaccine is widely available [52]. 

The current major therapeutic strategies for botulism are the human-derived BabyBIG [36] and an equine-derived HBAT [37]. These treatment options are either not widely available (BabyBIG) or may have potential side effects (HBAT). The HBAT contains fragments of IgG targeting seven BoNT types derived from equine plasma [37]. These fragments are pepsin digested IgG monomers and consist of < 2% intact IgG and ≥ 90% Fab or F(ab’)_2_ immunoglobulin fragments [53] in order to reduce the hypersensitivity reaction. Removing the Fc region of the IgG, which triggers the inflammatory side effects, allows HBAT administration without a skin sensitivity test [54]. However, the remaining Fab and F(ab’)_2_ fragments are cleared from the blood circulation more rapidly than intact IgGs [53], which makes HBAT plasma half-life shorter [53,54]. A short half-life in plasma may have serious consequences in the context of a massive outbreak of botulism or if the course of botulism or exposure to BoNT is prolonged, such as in intestinal or wound botulism. This occurred when a patient with BoNT/F intestinal botulism showed improvement after HBAT administration, but when Fab/F(ab’)_2_ IgG fragments were cleared from the circulation, BoNT/F rebound and bilateral descending flaccid paralysis recurred [54].

In conclusion, our newly-developed, humanized, novel IgG hu8ELC18 targeting the LC of BoNT/E protected mice against 5 MLD at a concentration as low as 2.5 ng/mouse in mouse protection model, and 8 ng protected against 1 LD50 of BoNT/E in mouse paralysis assays. IgG hu8ELC18 is expected to have a good tolerance in humans, can be produced in high quantities with a good yield in animal-free system and could, therefore, serve as a better therapeutic option with regards to tolerance in order to treat human botulism compared to HBAT. Additionally, the protective potential demonstrated by hu8ELC18 indicates that this IgG may be further developed into a short-term prophylactic product to protect personnel and population under bioterrorism risk prior to potential exposure to BoNT/E. This antibody, when combined with previously reported four antibodies against BoNT/A and BoNT/B [47] is expected to provide promising lead candidate for further development of an oligococktail of protective recombinant super-humanized IgGs against all three major botulinum toxins.

## 4. Materials and Methods

### 4.1. Ethical Statement and Animal Care

All animal experiments were conducted by expert veterinary surgeons under license (National Animal Experiment Board in Finland (ESAVI)/4135/04.10.07/2013). The female NMRI mice (20–22 g body weight) (Harlan Laboratories, Horst, The Netherlands) used in the experiments were kept in a separate laboratory designated only for use with mouse experiments at a temperature of 20 ± 2 °C and a relative humidity of 50%–60%. The mice were placed in socially-enriched cages (eight animals per cage) and fed ad libitum with commercial pellets. The well-being of the animals was monitored by the responsible veterinary surgeons at least once a day. The mice were monitored every hour for the first 5 h and additional monitoring was performed three times during the first day of the experiment. The monitoring was continued until the end of the experiments (96 h) (1–2 monitorings /day after the first 24 h). The mice with botulism symptoms were assessed by expert veterinary surgeons and euthanized by intraperitoneal (i.p.) injection of pentobarbital (200 mg/kg) at the humane end point. The arrival date of the mice, times of treatment, times and description of occurring symptoms, and times of euthanasia were recorded.

The in vivo paralysis assay was performed using an approved procedure included in the UK Home Office project license (PPL#80/2634, granted to Dr. Sesardic, Division of Bacteriology, National Institute for Biological Standards and Control (NIBSC), a centre of Medicine and Healthcare products Regulatory Agency). The procedure involves administration subcutaneously into mice of a sub-lethal dose of botulinum toxin premixed with antibody for neutralization studies, and observation for signs of abdominal ptosis with local palsy in mice over a period of 24 h. No animals experience systemic botulinum toxicity in this procedure and the test is terminated after 24 h. All experiments comply with the UK Home Office regulations for the use of animals in research under the Animals (Scientific Procedures) Act 1986 (ASPA) and revised European Directive 2010/63 EU on the protection of animals. Experiments performed at NIBSC for this study were approved by the local animal research oversight committee AWERB (Animal Welfare and Ethics Review body (Home Office project license PPL #80/2634. Date of approval 5 November 2012).

### 4.2. Humanization of scFv ELC18 by Germline-Humanization

The ELC18 sequence was aligned with the human germline genes using the IMGT/V-QUEST online tool from IMGT [55]. This tool allows the identification of the human germline genes most similar to any given variable region and the calculation of the GI, defined as the percentage identity between a given FR and the most similar human germline sequence. Based on the physiochemical classes of the AAs, differences in the FRs were classified as very similar, similar, dissimilar, and very dissimilar AA. First, humanized variants of the heavy chain variable segment (VH) and light chain variable segment (VL) were designed by exchanging the very similar AA and similar AA followed by dissimilar AA. The humanized VH and VL genes were then synthesized by GeneArt Gene Synthesis (Thermo Fisher, Langenselbold, Germany) and were used for expression of scFv-Fc. Altogether, eight humanized variants (hu1-8ELC18) in scFv-Fc format were constructed.

### 4.3. Production and Purification of scFv-Fc Antibodies

The production of ELC18 scFv-Fc was described previously [45]. Briefly, all humanized variants of ELC18 were sub-cloned into pCSE2.5-mIgG2c-Fc-XP and produced as scFv-Fc antibody in HEK293-6E cells (National Research Council, NRC), Biotechnological Research Institute, BRI) cultured in chemically defined medium, F17 (Thermo Fisher Scientifics, Waltham, MA, USA) supplemented with 1 g/L pluronic F68 (Applichem, Darmstadt, Germany), 4 mM l-glutamine (Biochrom GmbH, Berlin, Germany) and 25 mg/L G418 (Biochrom GmbH, Berlin, Germany), as previously described [56]. The scFv-Fc produced were chimeric macaque-mouse antibodies. DNA was used for the transient transfection of 25 mL cultures of HEK293-6E cells in 125 mL Erlenmeyer shake flasks. After 48 h of culture with shaking at 110 rpm in a Minitron orbital shaker (Infors GmbH, Einsbach, Germany) at 37 °C, under an atmosphere containing 5% CO_2_, one volume of culture medium, with a final concentration of 0.5% (*w*/*v*) tryptone N1 (TN1, Organotechnie S.A.S., La Courneuve, France), scFv-Fc were purified on a UNOsphere SUPrA column (Biorad, Hercules, CA, USA) with a Profinia apparatus (Biorad, Hercules, CA, USA), according to the manufacturer’s instructions.

### 4.4. ELISA Assay

ELISA was performed in 96-well microtiter plates (MaxiSorp, Nunc, Langenselbold, Germany. Each well was coated with BoNT/E (100 ng per well) (Metabiologics, Madison, WI, USA), in 100 μL of phosphate-buffered saline (PBS) by incubating overnight at 4 °C. The coated wells were washed three times with PBST (PBS + 0.05% Tween 20) with an ELISA washer (Tecan, Crailsheim, Germany). The wells were then blocked by incubation with 2% (*w*/*v*) skim milk powder in PBS, supplemented with 0.1% Tween 20 (2% M-PBST) for 1.5 h at room temperature, and then washed three times with PBST. For the antigen ELISA, the humanized variants of ELC18 were diluted in 100 μL of 2% M-PBST and incubated in the antigen-coated wells for 1.5 h at room temperature. The wells were then washed three times with PBST. Bound scFv-Fc was detected with a peroxidase-labeled goat anti-mouse antibody recognizing the murine part of the Fc fragment (Sigma-Aldrich, Munich, Germany A0168), visualized in a detection reaction with 3,3′,5,5′-tetramethylbenzidine (TMB) as substrate. The staining reaction was stopped by adding 100 μL of 1 N sulfuric acid. Absorbance at 450 nm (reference wavelength: 620 nm) was measured in a SUNRISE microtiter plate reader (Tecan, Crailsheim, Germany).

### 4.5. Cloning of Germline-Humanized IgG hu8ELC18

The germline-humanized hu8ELC18 full length IgG was constructed and expressed as described previously [57]. Briefly, the DNA sequences for the variable and constant regions of the heavy and light chains were obtained by PCR and sub-cloned sequentially by infusion into HKgenEFss vector. This generic vector was internally developed for optimal expression into the rat myeloma cell line YB2/0. Bacterial transformation was performed in *Escherichia coli* Top10 cells (Thermo Fisher, C4040) for each plasmid ligation in order to select the ligated product and amplification. Positive clones were selected by PCR and digestion of individual colonies was performed. Subsequently, colonies were grown overnight at 37 °C in LB medium with ampicillin. Plasmids were isolated using the NucleoSpin kit (Macherey-Nagel, Düren, Germany), and inserted sequences were verified (MWG, Ebersberg, Germany).

### 4.6. Production and Purification of IgG hu8ELC18

The stable expression of the humanized antibody was obtained as described previously [57]. Briefly, YB2/0 cells (ATCC CRL1662) were stably transfected with the linearized expression vectors. The hu8ELC18 antibody was produced in YB2/0 over 5–7 days using EMS (Invitrogen, Villebon Sur Yvette, France), 5% ultra low IgG FCS (PAA) and 0.5 g/L G418. The monoclonal hu8ELC18 was purified from culture supernatant by affinity chromatography onto protein A sepharose (GE-Healthcare, Vélizy-Villacoublay, France, 28-9365-47). The level of aggregates and endotoxins were determined by gel filtration on Superdex HR/200 (GE-Healthcare, 17-5175-01) and by LAL (limulous amoebocyte lysate) testing [58], respectively, and were always below 2%. Antibody quality and purity was also controlled by SDS-PAGE and Coomassie staining. In addition, glycosylation patterns and the core fucose percentage were determined for each purified antibody by high performance capillary electrophoresis laser induced fluorescence (HPCE-Lif) [59,60] confirming the characteristic with EMABling platform (LFB, Alès, France).

### 4.7. BoNT Toxin Complex Preparation

The BoNT/E producing *C. botulinum* strain, CB-M3 was pre-inoculated in 10 mL of deoxygenated tryptone-peptone-glucose-yeast extract (TPGY) broth (50 g/L of tryptone, 5g/L of peptone, 20 g/L of yeast extract (Difco, Sparks, MD, USA, 211699, 211820, 211931), 4 g/L of glucose, (VWR, Helsinki, Finland, 50-99-7), and 1 g/L of sodium thioglycolate (Merckmillipore, Darmstadt, Germany, 106691) from spore stock and grown for 24 h at 30 °C. The culture was grown in an anaerobic work station (MK III, Don Whitley Scientific, Ltd., Shipley, UK) under atmospheric conditions of 85% N_2_, 10% CO_2_, and 5% H_2_. The 5 mL of culture was used to inoculate 500 mL of deoxygenated TPGY and grown anaerobically for five days at 30 °C. The culture supernatant was collected by centrifugation, activated by trypsinization (200 μg/mL) (Sigma-Aldrich, T1426) followed by anti-trypsin treatment (400 μg/mL) (Sigma-Aldrich, A6150), aliquoted, and stored at −20 °C.

### 4.8. Toxicity Titration and Calibration of BoNT Complexes

To determine toxicity, BoNT/E complex was ten-fold serially diluted in phosphate buffer (pH 6.5, 0.2 g/mL gelatin) and a volume of 0.5 mL of each dilution was injected i.p. into two female, NMRI mice (20–22 g) (Harlan Laboratories, Horst, The Netherlands). The mice were observed for four days and symptoms (fuzzy hair, muscle weakness, wasp-like narrowed waist) were recorded. The highest dilution killing both mice was subsequently two-fold serially diluted and a volume of 0.5 mL of each dilution was injected i.p into four mice. The highest dilution killing all four mice was determined as containing 1 mouse lethal dose (MLD) of BoNT/E complex per 0.5 mL (1 MLD/0.5 mL).

### 4.9. Mouse Protection Assays

The in vivo protective capacity of the humanized IgG hu8ELC18 was determined in mouse bioassays. The trypsin-activated BoNT/E complex was diluted in phosphate buffer to a concentration leading to a toxin test dose of 5 MLD/mouse. The toxin test dose was incubated for 30 min at room temperature with different concentrations of the hu8ELC18. A volume of 0.5 mL of the incubated culture supernatant-hu8ELC18 mixture was injected i.p into two to four female NMRI mice (20–22 g) (Harlan Laboratories, Horst, Netherlands). The hu8ELC18 concentrations were 25 µg, 2.5 µg, and 0.25 µg (four mice per concentration), 0.025 µg, and 0.0025 µg/mouse (two mice per concentration). The mice were observed for four days.

### 4.10. Mouse Paralysis Assay

Neutralization activity of IgG hu8ELC18 was assessed in in vivo paralysis model with a range of concentrations of antibody (from 1.0 µg to 0.32 ng per dose) and pure BoNT/E3 toxin (Metabiologics, Madison, WI, USA, Lot # E062805-01, strain Alaska) at 1 LD50 (24 pg) per dose. Toxin was trypsinised prior to use. All dilutions were prepared in gelatin (0.2% *w*/*v*) phosphate (50 mM di-sodium hydrogen orthophosphate) buffer, (pH 6.5). Toxin-antibody mixtures were left to stand for 30 min at room temperature before injecting 0.1 mL subcutaneously into female MF1 strain of mice, weighing between 16–20 g (*n* = 4 per antibody dose or treatment). All injections were performed within 30 min and all the mice were injected in the left inguinocrural region. The animals were observed by at least three independent observers and scores made after visual inspection of the degree of local palsy, i.e., flaccid paralysis caused by chemodenervated muscle at the site of injection. Observations are reported as ranging from 0 (no signs of paralysis), 1 (limited, just detectable paralysis indicated by slight bulge at the site of injection covering an area of approximately 0.5 cm or less), 2 (moderate pronounced bulge, covering an area greater than 0.5 cm diameter), or 3 (substantial, more extensive bulge extending over a large area and below hips/top of the thigh when viewed from the side). The positive control group of mice were injected with BoNT/E3 toxin alone, and the negative control group of mice were injected with 1.0 µg of antibody used in the assay in the absence of toxin [61].

### 4.11. Prophylaxis Assay

To test the prophylactic capacity of IgG hu8HLC18, 25 µg of hu8HLC18 was administered i.p. 1 d, 3 d, 7 d, and 14 d before BoNT/E complex administration into four female NMRI mice each (20–22 g) (Harlan Laboratories, Horst, Netherlands). A test dose of 5 MLD/mouse BoNT/E complex was administered i.p. after the given time to each animal. The mice were observed for 4 days and survival was assessed.

## Figures and Tables

**Table 1:** Localization and evaluation of amino acid (AA) differences between ELC18 framework regions (FR), those coded by ELC18 most similar human germline genes, and Germinality Index (GI) of different FRs.

Framework Region		Total Number of AAs	Number of Identical AAs	Number of Very Similar AAs	Number of Similar AAs	Number of Dissimilar AAs	Number of Very Dissimilar AAs
VH							
	FR1-IMGT	25	20	0	2	2	1
	FR2-IMGT	17	16	0	0	1	0
	FR3-IMGT	38	33	0	2	2	1
	FR4-IMGT	11	9	0	0	1	1
	FR-IMGT	91	78	0	4	6	3
	GI	87.5%	100%	0%	4.4%	6.6%	3.3%
VL							
	FR1-IMGT	26	23	1	1	0	1
	FR2-IMGT	17	15	1	0	1	0
	FR3-IMGT	36	33	0	0	2	1
	FR4-IMGT	10	9	0	1	0	0
	FR-IMGT	89	80	2	2	3	2
	GI	89.9%	100%	2.2%	2.2%	3.4%	2.2%
VH and VL		180	158	2	6	9	5
	GI	87.8%	100%	1.1%	3.3%	5.0%	2.8%

**Table 2:** Humanized variants of ELC18 and their corresponding Germinality Index (GI).

Variant	VH Variant	VL Variant	Total GI
ELC18	Macaque VH	Macaque VL	87.8%
hu1ELC18	Macaque VH	hu1VL	90.1%
hu2ELC18	Macaque VH	hu2VL	91.8%
hu3ELC18	hu1VH	Macaque VL	90.0%
hu4ELC18	hu1VH	hu1VL	92.3%
hu5ELC18	hu1VH	hu2VL	94.0%
hu6ELC18	hu2VH	Macaque VL	93.3%
hu7ELC18	hu2VH	hu1VL	95.6%
hu8ELC18	hu2VH	hu1VL	97.3%

**Table 3:** Results of in vivo protection assays against 5 mouse lethal dose (MLD) BoNT/E complex.

hu8ELC18 IgG Concentration (µg)	Number of Survived/Total Mice
25	4/4
2.5	4/4
0.25	4/4
0.025	2/2
0.0025	2/2
0	0/4

**Table 4:** Prophylaxis assay of 25 µg/mice hu8ELC18 administration (i.p.) followed by injection of 5 mouse lethal dose (MLD) of BoNT/E complex (i.p.) after 1, 3, 7, and 14 days.

BoNT/E Injection after hu8ELC18 Administration (Days)	Number of Survived Mice/Total Mice
1	3/3
3	4/4
7	4/4
14	4/4
Control (no antibody)	0/4

## References

[1] Arnon S.S., Schechter R., Inglesby T.V., Henderson D.A., Bartlett J.G., Ascher M.S., Eitzen E., Fine A.D., Hauer J., Layton M. (2001). Botulinum toxin as a biological weapon: Medical and public health management. JAMA.

[2] Sesardic D., Jones R.G., Leung T., Alsop T., Tierney R. (2004). Detection of antibodies against botulinum toxins. Mov. Disord..

[3] Hatheway C.L., Hauschild A.H.W., Doods K.L. (1993). *Clostridium botulinum* and other clostridia that produce botulinum neurotoxin. Clostridium botulinum, Ecology and Control in Foods.

[4] Lindström M., Korkeala H. (2006). Laboratory diagnostics of botulism. Clin. Microbiol. Rev..

[5] Peck M.W. (2009). Biology and genomic analysis of *Clostridium botulinum*. Adv. Microb. Physiol..

[6] Hill K.K., Smith T.J., Rummel A., Binz T. (2013). Genetic diversity within *Clostridium botulinum* serotypes, botulinum neurotoxin gene clusters and toxin subtypes. Botulinum Neurotoxins.

[7] Barash J.R., Arnon S.S. (2014). A novel strain of *Clostridium botulinum* that produces type B and type H botulinum toxins. J. Infect. Dis..

[8] Dover N., Barash J.R., Hill K.K., Xie G., Arnon S.S. (2014). Molecular characterization of a novel botulinum neurotoxin type H gene. J. Infect. Dis..

[9] Maslanka S.E., Luquez C., Dykes J.K., Tepp W.H., Pier C.L., Pellett S., Raphael B.H., Kalb S.R., Barr J.R., Rao A. (2015). A novel botulinum neurotoxin, previously reported as serotype H, has a hybrid-like structure with regions of similarity to the structures of serotypes A and F and is neutralized with serotype A antitoxin. J. Infect. Dis..

[10] Chen Y., Korkeala H., Aarnikunnas J., Lindström M. (2007). Sequencing the botulinum neurotoxin gene and related genes in *Clostridium botulinum* type E strains reveals *orfx3* and a novel type E neurotoxin subtype. J. Bacteriol..

[11] Couesnon A., Raffestin S., Popoff M.R. (2006). Expression of botulinum neurotoxins A and E, and associated non-toxin genes, during the transition phase and stability at high temperature: Analysis by quantitative reverse transcription-PCR. Microbiology.

[12] Froude J.W., Stiles B.G., Pelat T., Thullier P. (2011). Antibodies for biodefense. mAbs.

[13] Rosenau W. (2001). Aum shinrikyo’s biological weapons program: Why did it fail?. Stud. Confl. Terror..

[14] Sakaguchi G. (1982). *Clostridium botulinum* toxins. Pharmacol. Ther..

[15] Popoff M.R., Bouvet P. (2013). Genetic characteristics of toxigenic clostridia and toxin gene evolution. Toxicon.

[16] Yokosawa N., Tsuzuki K., Syuto B., Oguma K. (1986). Activation of *Clostridium botulinum* type E toxin purified by two different procedures. J. Gen. Microbiol..

[17] Pellizzari R., Rossetto O., Schiavo G., Montecucco C. (1999). Tetanus and botulinum neurotoxins: Mechanism of action and therapeutic uses. Philos Trans. R. Soc. Lond. B Biol. Sci..

[18] Montecucco C. (1986). How do tetanus and botulinum toxins bind to neuronal membranes?. Trends Biochem. Sci..

[19] Humeau Y., Doussau F., Grant N.J., Poulain B. (2000). How botulinum and tetanus neurotoxins block neurotransmitter release. Biochimie.

[20] Simpson L.L. (2004). Identification of the major steps in botulinum toxin action. Annu. Rev. Pharmacol. Toxicol..

[21] Schiavo G., Matteoli M., Montecucco C. (2000). Neurotoxins affecting neuroexocytosis. Physiol. Rev..

[22] Elias M., Al-Saleem F., Ancharski D.M., Singh A., Nasser Z., Olson R.M., Simpson L.L. (2011). Evidence that botulinum toxin receptors on epithelial cells and neuronal cells are not identical: Implications for development of a non-neurotropic vaccine. J. Pharmacol. Exp. Ther..

[23] Pirazzini M., Tehran D.A., Zanetti G., Lista F., Binz T., Shone C.C., Rossetto O., Montecucco C. (2015). The thioredoxin reductase-Thioredoxin redox system cleaves the interchain disulphide bond of botulinum neurotoxins on the cytosolic surface of synaptic vesicles. Toxicon.

[24] Jones R., Ochiai M., Liu Y., Ekong T., Sesardic D. (2008). Development of improved SNAP25 endopeptidase immuno-assays for botulinum type A and E toxins. J. Immunol. Methods.

[25] Leclair D., Farber J.M., Doidge B., Blanchfield B., Suppa S., Pagotto F., Austin J.W. (2013). Distribution of *Clostridium botulinum* type E strains in Nunavik, Northern Quebec, Canada. Appl. Environ. Microbiol..

[26] Hielm S., Hyytiä E., Andersin A., Korkeala H. (1998). A high prevalence of *Clostridium botulinum* type E in Finnish freshwater and Baltic Sea sediment samples. J. Appl. Microbiol..

[27] Hyytiä E., Hielm S., Björkroth J., Korkeala H. (1999). Biodiversity of *Clostridium botulinum* type E strains isolated from fish and fishery products. Appl. Environ. Microbiol..

[28] Hielm S., Björkroth J., Hyytiä E., Korkeala H. (1998). Prevalence of *Clostridium botulinum* in Finnish trout farms: Pulsed-field gel electrophoresis typing reveals extensive genetic diversity among type E isolates. Appl. Environ. Microbiol..

[29] King L.A., Niskanen T., Junnikkala M., Moilanen E., Lindström M., Korkeala H., Korhonen T., Popoff M., Mazuet C., Callon H. (2009). Botulism and hot-smoked whitefish: A family cluster of type E botulism in France, September 2009. Euro Surveill..

[30] Lindström M., Vuorela M., Hinderink K., Korkeala H., Dahlsten E., Raahenmaa M., Kuusi M. (2006). Botulism associated with vacuum-packed smoked whitefish in Finland, June–July 2006. Euro Surveill.

[31] Mazuet C., Sautereau J., Legeay C., Bouchier C., Bouvet P., Popoff M.R. (2015). An atypical outbreak of food-borne botulism due to clostridium botulinum types B and E from ham. J. Clin. Microbiol..

[32] Simpson L.L., DasGupta B.R. (1983). Botulinum neurotoxin type E: Studies on mechanism of action and on structure-activity relationships. J. Pharmacol. Exp. Ther..

[33] Arnon S.S., Rood J., Mc Clane B., Songer J., Titball R. (1997). Human Tetanus and Human Botulism. Clostridia: Molecular Biology and Pathogenesis.

[34] Cherington M. (1998). Clinical spectrum of botulism. Muscle Nerve.

[35] Meunier F.A., Schiavo G., Molgó J. (2002). Botulinum neurotoxins: From paralysis to recovery of functional neuromuscular transmission. J. Physiol. Paris.

[36] Arnon S.S., Schechter R., Maslanka S.E., Jewell N.P., Hatheway C.L. (2006). Human botulism immune globulin for the treatment of infant botulism. N. Engl. J. Med..

[37] Centers for Disease Control and Prevention (CDC) (2010). Investigational heptavalent botulinum antitoxin (HBAT) to replace licensed botulinum antitoxin AB and investigational botulinum antitoxin E. MMWR Morb. Mortal. Wkly. Rep..

[38] Black R.E., Gunn R.A. (1980). Hypersensitivity reactions associated with botulinal antitoxin. Am. J. Med..

[39] Hibbs R.G., Weber J.T., Corwin A., Allos B.M., Abd el Rehim M.S., Sharkawy S.E., Sarn J.E., McKee K.T. (1996). Experience with the use of an investigational F(ab’)2 heptavalent botulism immune globulin of equine origin during an outbreak of type E botulism in Egypt. Clin. Infect. Dis..

[40] Pelat T., Thullier P. (2009). Non-human primate immune libraries combined with germline humanization: An (almost) new and powerful approach for the isolation of therapeutic antibodies. mAbs.

[41] Tan P., Mitchell D.A., Buss T.N., Holmes M.A., Anasetti C., Foote J. (2002). “Superhumanized” antibodies: Reduction of immunogenic potential by complementarity-determining region grafting with human germline sequences: Application to an anti-CD28. J. Immunol..

[42] Williams G.T., Jolly C.J., Köhler J., Neuberger M.S. (2000). The contribution of somatic hypermutation to the diversity of serum immunoglobulin: Dramatic increase with age. Immunity.

[43] Pelat T., Bedouelle H., Rees A.R., Crennell S.J., Lefranc M., Thullier P. (2008). Germline humanization of a non-human primate antibody that neutralizes the anthrax toxin, by in vitro and in silico engineering. J. Mol. Biol..

[44] Robert R., Streltsov V.A., Newman J., Pearce L.A., Wark K.L., Dolezal O. (2010). Germline humanization of a murine Aβ antibody and crystal structure of the humanized recombinant Fab fragment. Protein Sci..

[45] Miethe S., Rasetti-Escargueil C., Avril A., Liu Y., Chahboun S., Korkeala H., Mazuet C., Popoff M.R., Pelat T., Thullier P. (2015). Development of human-like scFv-fc neutralizing botulinum neurotoxin E. PLoS ONE.

[46] Rosenwasser L.J., Busse W.W., Lizambri R.G., Olejnik T.A., Totoritis M.C. (2003). Allergic asthma and an anti-CD23 mAb (IDEC-152): Results of a phase I, single-dose, dose-escalating clinical trial. J. Allergy Clin. Immunol..

[47] Miethe S., Mazuet C., Liu Y., Tierney R., Rasetti-Escargueil C., Avril A., Frenzel A., Thullier P., Pelat T., Urbain R. (2016). Development of germline-humanized antibodies neutralizing botulinum neurotoxin A and B. PLoS ONE.

[48] Byrd J.C., O’Brien S., Flinn I.W., Kipps T.J., Weiss M., Rai K., Lin T.S., Woodworth J., Wynne D., Reid J. (2007). Phase 1 study of lumiliximab with detailed pharmacokinetic and pharmacodynamic measurements in patients with relapsed or refractory chronic lymphocytic leukemia. Clin. Cancer Res..

[49] Hust M., Meyer T., Voedisch B., Rülker T., Thie H., El-Ghezal A., Kirsch M.I., Schütte M., Helmsing S., Meier D. (2011). A human scFv antibody generation pipeline for proteome research. J. Biotechnol..

[50] Thyagarajan B., Gopalakrishnakone P., Balali-Mood M., Ram Singh B., Llewellyn L. (2015). Antidotes to botulinum neurotoxin. Biological Toxins and Bioterrorism.

[51] Smith L.A. (2009). Botulism and vaccines for its prevention. Vaccine.

[52] Rusnak J.M., Smith L.A. (2009). Botulinum neurotoxin vaccines: Past history and recent developments. Hum. Vaccines.

[53] Foster K.A. (2014). Molecular Aspects of Botulinum Neurotoxin.

[54] Fagan R.P., Neil K.P., Sasich R., Luquez C., Asaad H., Maslanka S., Khalil W. (2011). Initial recovery and rebound of type F intestinal colonization botulism after administration of investigational heptavalent botulinum antitoxin. Clin. Infect. Dis..

[55] IMGT/V-QUEST Online Tool. http://www.imgt.org.

[56] Jäger V., Büssow K., Wagner A., Weber S., Hust M., Frenzel A., Schirrmann T. (2013). High level transient production of recombinant antibodies and antibody fusion proteins in HEK293 cells. BMC Biotechnol..

[57] Sibéril S., Dutertre C., Boix C., Bonnin E., Ménez R., Stura E., Jorieuxb S., Fridmana W., Teillaud J. (2006). Molecular aspects of human FcγR interactions with IgG: Functional and therapeutic consequences. Immunol. Lett..

[58] Tsuji K., Steindler K.A., Harrison S.J. (1980). Limulus amoebocyte lysate assay for detection and quantitation of endotoxin in a small-volume parenteral product. Appl. Environ. Microbiol..

[59] Guttman A. (1995). On the separation mechanism of capillary sodium dodecyl sulfate-gel electrophoresis of proteins. Electrophoresis.

[60] Szabo Z., Guttman A., Bones J., Karger B.L. (2011). Rapid high-resolution characterization of functionally important monoclonal antibody *N*-glycans by capillary electrophoresis. Anal. Chem..

[61] Jones R.G., Alsop T.A., Hull R., Tierney R., Holley J., Sesardic D. (2006). Botulinum type A toxin neutralisation by specific IgG and its fragments: A comparison of mouse systemic toxicity and local flaccid paralysis assays. Toxicon.

